# Medical Ultrasound in the Evaluation of the Carpal Tunnel: A Critical Review

**DOI:** 10.7759/cureus.3487

**Published:** 2018-10-23

**Authors:** Georgi P Georgiev, Vesselin Karabinov, Georgi Kotov, Alexandar Iliev

**Affiliations:** 1 Orthopaedics and Traumatology, Queen Giovanna Hospital, Sofia, BGR; 2 Neurology, National Cardiology Hospital, Sofia, BGR; 3 Anatomy, Histology and Embryology, Medical University of Sofia, Sofia, BGR

**Keywords:** medical ultrasound, sonography, carpal tunnel, review

## Abstract

The use of medical ultrasound as an imaging modality in the evaluation of the carpal tunnel syndrome has been growing. Technical progress and the introduction of new, high-quality devices and ultra-high frequency transducers with smaller dimensions have led to a better quality of acquired images and the development of new examination methodologies. The sonographic method has a number of advantages: available, non-invasive, cheaper, and allows an easier and faster evaluation and diagnosis of carpal tunnel syndrome, along with the possibility to assess various parameters of the median nerve, including its size, blood flow, and mobility. In addition, medical ultrasound may be used to detect anatomical variations and space-occupying lesions in the carpal tunnel to monitor the therapeutic response in patients suffering from carpal tunnel syndrome and to evaluate median nerve injuries. Herein, we present a review of the pertinent literature with regard to the diagnostic potential of medical ultrasound in the evaluation of pathological changes in the carpal tunnel.

## Introduction and background

Medical ultrasound (MUS) is a diagnostic imaging modality used increasingly more often in everyday medical practice. The sonographic examination makes it possible to visualize different structures with a resolution of up to 0.1 mm in a horizontal and 0.2 mm in a vertical plane. It acquires a multi-layered image of the examined area and is practically the only imaging modality that allows a dynamic examination. In theory, MUS is harmless for the patient and represents a non-invasive method, free of ionizing radiation, conducted through portable devices. It may be used for multiple evaluations, which makes it possible to trace the findings on time. Last but not least, its price is relatively low. Technical progress and the introduction of new high-quality devices and ultra-high frequency transducers with smaller dimensions have led to a better quality of the acquired images and the development of new examination methodologies. This, in turn, is a prerequisite for a wider use of MUS for examinations of the wrist and the hand, as well as the carpal tunnel (CT) in particular [1-4].

The aim of the present manuscript was to make a review of the pertinent literature with regard to the diagnostic potential of MUS in the evaluation of pathological changes in the CT.

## Review

Anatomy of the carpal tunnel

The carpal tunnel (CT) is bounded by the bones of the wrist (the hook of the hamate bone, the triquetrum, and the pisiform bone on the medial side; the scaphoid and the trapezium bone on the lateral side), and the flexor retinaculum. The tendons of the flexor muscles and the median nerve pass through it. The walls of the tunnel are formed by the superficial thick layer of the flexor retinaculum to the front and by the deep layer of the retinaculum and the bones of the wrist to the back. The tendons of the flexor digitorum superficialis and the flexor digitorum profundus are found in the medial part of the tunnel. The tendon of the flexor pollicis longus is located laterally to the flexor digitorum profundus, with the median nerve passing superficially [5].

Different anatomical variations, such as those of the palmaris longus (accessory tendons, “reversed” palmaris longus, palmaris profundus muscle), persistent median artery, “bifid” median nerve and others, are a prerequisite for the development of a condition known as carpal tunnel syndrome (CTS) [6]. Most often, the CTS is an idiopathic condition but may also be the result of a traumatic injury, such as a fracture of the distal radius or a dislocation of the carpal bones, as well as a consequence of rheumatoid arthritis, hypothyroidism, oral contraceptives, diabetes mellitus, acromegaly, or pregnancy [1,7].

Sonographic examination of patients with carpal tunnel syndrome

The use of MUS as an imaging modality in the evaluation of CTS has been growing. MUS in patients with the electromyography (EMG) findings of CTS has the advantage of revealing structural changes and nerve swelling, but it can also be used to visualize other pathologies, which cannot be detected through electrophysiological examinations (muscle hypotrophy, anatomical variants, tenosynovitis, tumors, etc.). Moreover, additional findings reported through MUS, such as the echogenicity of the median nerve, its mobility, or an edema of the flexor retinaculum, may also aid in the diagnosis of CTS. The sonographic findings in patients suffering from CTS include median nerve swelling, compression of the nerve, palmar migration, and thickening of the flexor retinaculum, along with changes in the characteristic features of the median nerve [8-10].

The sonographic method of examination has a number of advantages: available, non-invasive, cheaper, and allows an easier and faster evaluation and diagnosis of CTS, along with the possibility to assess various parameters of the median nerve, including its size, blood flow (through Doppler), and mobility (through dynamic ultrasound) [9,11]. The median nerve can be clearly visualized through MUS on a transverse section as a zone of hypoechoic nerve fibers with hyperechoic endings, located immediately above the flexor tendons and below the hyperechoic flexor retinaculum, which covers the nerve upon its passing through the CT [9].

The sonographic examination is conducted with an 8-14 MHz or a 6-18 MHz linear transducer. Patients are positioned with their face turned towards the physician, their wrists resting on a plain surface, with their forearms in the supine position and their fingers – slightly flexed. Two methods are used to measure the cross-sectional area (CSA) of the median nerve. In the first, a continuous line is traced along the internal hyperechoic edge of the nerve; the CSA of the circled area is then calculated through computerized software. The second approach is based on the measurement of the anteroposterior and transverse diameter of the nerve. The CSA of the ellipse is then calculated from the values of these parameters through a mathematical formula. There is a certain level of correlation between the results obtained through the two methods. Measurements of the median nerve of an amputated limb made through the two methods using MUS were compared to those directly obtained by measuring the CSA on a frozen segment. The correlation was 0.982 for the first method (direct tracing) and 0.992 for the second method (anteroposterior and transverse measurement) [12]. The inter-reader reliability when measuring the CSA of the nerve at the tunnel inlet using the method of direct tracing and the method of the ellipse is fairly good, with correlation coefficients of 0.81 and 0.97, respectively. On the other hand, the reliability when measuring the CSA of the nerve at the tunnel outlet is low, probably due to the orientation of the nerve at the outlet, where it is situated in a more dorsal position. This makes good visualization and proper measurements more difficult [13].

The CSA is the most important parameter of the median nerve, evaluated through MUS. Measurements exceeding 10 mm^2^ have diagnostic significance for CTS [14]. Another important parameter is the compression of the median nerve, which may be observed at the point where the nerve passes below the flexor retinaculum. Compression of the nerve may be described as a globular swelling. The sensitivity of this symptom has not been fully established; however, its specificity varies between 95.8% and 100% [15]. The third parameter is the increased intraneural blood flow. It can be observed on В-mode images or, even better, through color Doppler, with the accuracy of detection of CTS reaching 91% [16]. The hypervascularity and hypoechogenicity of the nerve are observed together with a greater CSA; in fact, there is a 90% probability of CTS being present if all three of these parameters are positive, even in cases where the electrophysiological examination may be normal [17].

Klauser et al. studied the use of an additional measurement of the CSA at a proximal site for the calculation of a new parameter – the difference between the proximal CSA and CSA measured at the level of the CT [14]. According to various sources, the mean normal CSA of the median nerve varies between 6.1 and 10.4 mm^2^ and the difference between these two values (4.3 mm^2^) represents 51% of the normal median nerve CSA [18]. The threshold values vary between 9 and 14 mm^2^ and the difference between these two values (5 mm^2^) represents 59% of the normal values (8.4 mm^2^) [18]. Klauser et al. suggested that comparing the degree of median nerve swelling in the CT with the CSA of the nerve measured proximally would compensate for the interindividual variability of the median nerve CSA and contribute to a more accurate diagnosis of CTS [14].

Hobson-Webb et al. calculated the ratio between the CSA of the median nerve at the level of the wrist and the CSA measured approximately 12 cm proximally from the wrist and established the wrist-to-forearm ratio (WFR) [19]. In their study, they included 44 patients and 18 controls and found that the diagnosis of CTS improved when using the WFR as opposed to a diagnosis established only on the basis of measuring the CSA at the level of the wrist. WFR was 2.1 ±0.5 in patients presenting with CTS and 1.0 ± 0.1 in asymptomatic volunteers. A WFR of 1.4 or more gave 100% sensitivity for detecting patients suffering from CTS.

Fu et al. evaluated the diagnostic significance of the inlet-to-outlet median nerve CSA ratio (IOR) in patients with a clinical and electrophysiological diagnosis of CTS [20]. These authors studied 46 patients with clinical and electrophysiological findings consistent with a diagnosis of CTS and 44 healthy volunteers. CSA was measured at the inlet and outlet of the CT and IOR was calculated for each wrist. The mean inlet CSA was 8.7 mm^2^ in healthy individuals and 14.6 mm^2^ in patients with CTS. The mean IOR was 1.0 in the control group and 1.6 in those suffering from CTS. This study reported the diagnostic advantage of using the parameter IOR instead of mean inlet CSA. An IOR cut-off value of 1.3 or more gave 93% specificity and 91% sensitivity in the diagnosis of CTS.

Different anatomical variations in the CT have been discovered through a sonographic examination. These include a persistent median artery, a bifid median nerve, variations of the motor branch and the palmar cutaneous branch of the median nerve, a reversed palmaris longus muscle, Martin-Gruber anastomosis, Limburg-Comstock syndrome, etc. [5]. Although mostly asymptomatic, these variations may sometimes mimic the clinical symptoms of CTS. Cartwright et al. studied 1026 wrists of individuals who worked full-time in manual labor positions and reported that the incidence of anatomical variations of muscles at the level of the CT was higher in workers suffering from CTS than healthy ones [11]. MUS revealed hypertrophy of the lumbrical muscles or the flexor digitorum, as well as muscle intrusion in the CT. In patients with diabetic neuropathy, a sonographic examination revealed diffuse thickening of the peripheral nerves, with a significantly higher CSA of the median and the ulnar nerve compared to those of matched controls [21]. In patients with CTS, the authors reported CSA of the median nerve 13.9 mm^2^ compared to only 7.89 mm^2^ in control individuals and further established a significant difference in the WFR [21].

Previous studies on the use of MUS in CTS, which used EMG as the reference standard revealed that the sensitivity of the method was 82%-94% and its specificity was 65%-97% [14]. The relatively large interval of the indicated percentages of sensitivity and specificity contributes to the variety of opinions on the benefits of MUS in the diagnosis of CTS. The sensitivity of the sonographic examination may be improved if the diagnostic criteria combine several different parameters. Chan et al. concluded that combined measurements of the CSA of the median nerve at two levels (proximal to the CT inlet and at the CT inlet) yielded higher sensitivity and specificity than any of them considered alone [22]. Nakamichi and Tachibana measured the mean CSA of the median nerve in the CT (average of the CSA measured at three separate levels: proximal, mid, and distal CT), thus improving sensitivity from 43% to 67% [12]. In another study, the CSA measured in the proximal CT was 11 mm^2^ and revealed very high specificity (98%), yet a large number of false negative results (26.4%). When combining this measurement with the sonographic signs of compression on longitudinal scans, the false negative results decreased to 10.9% [23].

In the study of Ooi et al., patients were examined through MUS and EMG [24]. The aim of this study was to evaluate the diagnostic performance of grayscale, color Doppler, and dynamic ultrasound in the diagnosis of CTS while using EMG as the reference standard. Secondly, this study aimed at assessing the correlation between the CSA of the median nerve and the severity of the CTS as determined through EMG. In addition, the authors tried to evaluate the reliability of MUS in patients suffering from CTS. Fifty-one patients were studied. A statistical analysis revealed a directly proportional correlation between the CSA of the median nerve at the level of the pisiform bone and the duration of symptoms. No correlation was established between the sonographic findings and the chronic stage of the disease. Through MUS, a bifid median nerve was observed in four patients while in 3 others, the main pathological factor was tenosynovitis with degenerative tophi. The ability of the sonographic method to present the morphology of the median nerve and the surrounding structures may help avoid misdiagnosis in the case of tumors, which may simulate CTS, especially in patients with atypical EMG findings. The authors stated that measuring the CSA through MUS is an accurate method for diagnosing CTS and provides additional information for the degree of median nerve damage.

MUS is also a very accurate method in the diagnosis of severe cases of CTS, where EMG has lower accuracy [22]. A correlation exists between the CSA of the median nerve at the inlet of the CT and the mean distal motor latency. A greater latency is a marker for nerve demyelization at the level of the CT, which explains the changes observed with the sonographic examination. Nevertheless, as a whole, the CSA does not correlate well with the degree of symptom manifestation and the degree of functional impairment. Klauser et al. reported a high degree of accuracy when establishing the diagnosis of CTS though MUS [14]. Taking into account the non-invasive character of MUS as compared to EMG, a clinical evaluation together with a sonographic examination may prove to be the best possible approach. Using MUS together with an additional measurement of the nerve at the level of the pronator teres in order to monitor the therapeutic response needs to be assessed in future studies.

Role of sonographic examinations in monitoring therapeutic response in patients with CTS, median nerve injuries, and space-occupying lesions provoking CTS

MUS may be used for monitoring of the therapeutic response by measuring the decrease in the CSA of the median nerve after surgery or splinting, as well as after the administration of corticosteroid treatment [25-28].

There are very few data in the literature regarding the role of MUS in the postoperative period after the surgical decompression of the median nerve. It could be used in patients who suffer from constant symptoms or symptoms that persist after an intervention has been made, including in the case of iatrogenic nerve lesions and upon an assessment of the need of reintervention. If surgical decompression of the median nerve leads to a normal CSA, this may ease the selection of patients in which there are indications for a second surgery. If the nerve does not change morphologically after surgery, the sonographic examination would not aid in determining in which patients the nerve is still compressed and where decompression has been successfully achieved; however, it could evaluate the flexor retinaculum as a marker of decompression. It should be noted that clinical improvement might take place at a different pace from that seen with MUS and a longer follow-up period might be needed in order to establish a correlation [21,28].

In addition, MUS is important in patients with injuries in the region of the median nerve. The epineurium, in this case, would be easily visualized and the nerve injury could be classified as axonotmesis, partial or full tear. Should any posttraumatic complications occur, MUS would help discover a cicatrix, an epineurium which failed to reconnect, or a neuroma. In case of iatrogenic nerve lesions, such as the transsection of the muscle branches of the median nerve during an endoscopic surgery in patients with CTS, discovering such a nerve lesion is an indication for an immediate surgery [21].

MUS may be used for the detection of space-occupying lesions mimicking CTS, such as a ganglion, fibroma, tenosynovitis, and tumors involving the median nerve itself – most often schwannomas and, in rare cases, neurofibromas. When nerve sheath tumors are situated in the CT, they typically present as uninterrupted hypoechoic structures, protruding from the nerve from which they originate [29].

Limitations of the sonographic examination of the carpal tunnel

The sonographic examination is a subjective method and depends on the expertise of the physician and the type of the device. When the clinical diagnosis and the sonographic examination are inconclusive, a suggested approach may be for the physician to proceed to an EMG examination. More and more studies are being released, which confirm the benefit of newer ultrasound techniques, such as power Doppler, microvascular imaging, and elastography, in the evaluation of CTS, which may improve the specificity and sensitivity of the sonographic examination. A significant disadvantage of MUS is that it does not provide any information regarding the function of the nerve and, consequently, cannot be sensitive for changes like the EMG examination, although patients suffering from CTS are anyway usually followed based on clinical signs and symptoms.

## Conclusions

Nowadays, the sonographic examination of the CT is a widely used method in the practice of orthopaedists, rheumatologists, neurologists, and neurosurgeons. This method is cheap, consumes a relatively short amount of time, is well tolerated by patients, and makes it possible to diagnose structural anomalies. Its sensitivity and specificity are comparable to those of electrophysiological examinations conducted when CTS is suspected. MUS may be used as an additional preoperative examination and in patients whose condition does not improve after surgery due to CTS, as well as for the evaluation of injuries of the median nerve and space-occupying lesions.
